# Acceptability and Effectiveness of Low Intensity Mental Health Services for Children and Young People Attending a General Hospital

**DOI:** 10.1192/bjo.2024.157

**Published:** 2024-08-01

**Authors:** Anna Roach, Mike Groszmann, The UCLH Psychological Medicine Team, The Lucy Project Team

**Affiliations:** 1 UCL Great Ormond Street Institute of Child Health, London, United Kingdom; 2 University College Hospitals, London, United Kingdom

## Abstract

**Aims:**

Despite the high prevalence of mental health disorders in children and young people (CYP) with long-term health conditions (LTCs), these difficulties are often overlooked and untreated. Previous research demonstrated the effectiveness of low intensity psychological support provided via a drop-in mental health centre in a single specialist paediatric hospital. The aim of this study is to determine the effectiveness and acceptability of accessible low intensity mental health services for CYP attending a general hospital.

**Methods:**

This project was part of a wider prospective non-randomised single-arm multi-centre interventional study (Trial registration: ISRCTN15063954). CYP aged up to 25 years old with a LTC, who had been receiving care for their LTC for 6 months or more, and their parent/carer were eligible to be referred by their clinician or self-refer to the trial. The primary outcome is the difference in the total difficulties score on the Strengths and Difficulties Questionnaire (SDQ) reported by parent or CYP between baseline and 6 months. Interventions provided were: low intensity CBT, onward referral or signposting.

**Results:**

53 families were recruited at this hospital which made up 44% of the total study sample (120 families). Patients recruited were made up of 34 females, 18 males and one young person who identified as non-binary. The mean age of the CYP was 16.13 years and they were living with a range of different LTCs including cancer, asthma and diabetes. At baseline the average self-reported and parent reported SDQ scores were within the “very high” range (21.52 and 22.03, respectively). All participants were offered an initial assessment within 3 weeks of consenting (average 19.6 days) and treatment began within a month. Qualitative feedback from families has identified how the service “fills a gap” between physical and mental health and their satisfaction with how “time-sensitive” support was available.

**Conclusion:**

There is significant demand for this service and CYP living with different LTCs are accessing and utilising the service provided. This model of intervention allows timely access to evidence-based mental health support for CYP attending a general hospital for their physical health needs, compared with standard waiting times in other services.

